# Etiology of Inguinal Hernias: A Comprehensive Review

**DOI:** 10.3389/fsurg.2017.00052

**Published:** 2017-09-22

**Authors:** Stina Öberg, Kristoffer Andresen, Jacob Rosenberg

**Affiliations:** ^1^Center for Perioperative Optimization, Department of Surgery, Herlev Hospital, University of Copenhagen, Copenhagen, Denmark

**Keywords:** inguinal hernia, etiology, processus vaginalis, connective tissue alteration, risk factors, medial hernia, lateral hernia

## Abstract

**Background:**

The etiology of inguinal hernias remains uncertain even though the lifetime risk of developing an inguinal hernia is 27% for men and 3% for women. The aim was to summarize the evidence on hernia etiology, with focus on differences between lateral and medial hernias.

**Results:**

Lateral and medial hernias seem to have common as well as different etiologies. A patent processus vaginalis and increased cumulative mechanical exposure are risk factors for lateral hernias. Patients with medial hernias seem to have a more profoundly altered connective tissue architecture and homeostasis compared with patients with lateral hernias. However, connective tissue alteration may play a role in development of both subtypes. Inguinal hernias have a hereditary component with a complex inheritance pattern, and inguinal hernia susceptible genes have been identified that also are involved in connective tissue homeostasis.

**Conclusion:**

The etiology of lateral and medial hernias are at least partly different, but the final explanations are still lacking on certain areas. Further investigations of inguinal hernia genes may explain the altered connective tissue observed in patients with inguinal hernias. The precise mechanisms why processus vaginalis fails to obliterate in certain patients should also be clarified. Not all patients with a patent processus vaginalis develop a lateral hernia, but increased intraabdominal pressure appears to be a contributing factor.

## Introduction

Even though the lifetime risk of developing an inguinal hernia is 27% for men and 3% for women (1), the etiology remains uncertain. Inguinal hernias can be subdivided into lateral and medial hernias. Inguinal hernias are almost exclusively lateral in children (2), whereas women and men have both subtypes (3). Lateral hernias are more frequent, but medial hernias have a higher risk to recur after repair (4, 5). Lateral and medial hernias are often treated similarly, even though the described differences in age, sex, and recurrence rates imply different etiologies.

The aim was to summarize the evidence on hernia etiology, with focus on differences between lateral and medial hernias.

## Anatomy and Herniation

The inguinal canal starts at the internal inguinal ring and ends at the superficial ring, containing the spermatic cord in men and the round ligament in women. The integrity of the abdominal wall depends on the orientation of the inguinal canal, the transversalis fascia, and the sphincter-like function of the internal ring (6). A hypothesis to the high incidence of inguinal hernias in humans is that the abdominal wall was well constructed when we walked on four extremities, but that the groin area did not have enough time to adopt when we rose to standing on two legs. Lateral hernias arise from the internal inguinal ring, presumably through a patent processus vaginalis (7), and runs in the inguinal canal with or without exit through the superficial ring (8). Medial hernias herniate through a presumably weakened transversalis fascia in the Hesselbach’s triangle (8).

## Risk Factors for Developing a Primary Inguinal Hernia

The risk factors for developing an inguinal hernia can be divided into patient risk factors such as age and sex (9, 10), and external risk factors such as physically demanding work (11, 12).

### Patient Risk Factors

Risk factors for developing a primary inguinal hernia are male gender and old age (9, 10), a patent processus vaginalis (7), systemic connective tissue disorders (13, 14), and a low body mass index (BMI) (10, 15). Increasing age and low BMI increase the risk of both medial and lateral hernia repairs (12). However, high BMI increases the intraabdominal pressure (16) and also seems to increase the risk of developing a recurrence (4). The relationship probably has a risk of bias since it is easier to detect an inguinal hernia at lower BMI. Constipation does not appear to be a risk factor (17). Researchers have found an association to prostatic hypertrophy (17) but it is uncertain if it truly is a risk factor (18, 19).

### External Risk Factors

Smoking increases the risk of recurrence (4), but it is uncertain if it is a risk factor for developing a primary inguinal hernia (10, 12, 20). An explanation to a relationship between smoking and herniation could be increased collagen degradation and decreased synthesis, shown in human fibroblasts (21). High intraabdominal pressure is also proposed to be a risk factor (22). A database study on 1.5 million individuals showed an increased risk of primary lateral repairs with increased cumulative exposure to daily lifting and standing/walking (11), and by reducing standing/walking from ≥6 h to <4 h daily about 30% of primary lateral hernia repairs can be prevented (12). Intraabdominal pressure increases when coughing, jumping, etc. (16), and the relationship indicates that increased cumulative intraabdominal pressure is involved in lateral hernia formation (11, 12), probably through a patent processus vaginalis (23). For medial hernias, which lack a preformed defect, herniation was unaffected by increased cumulative work exposure (11, 12). Other factors that can increase the intraabdominal pressure, such as leisure-time physical activity and total load lifted per day, did not increase the risk of neither lateral nor medial repairs (12). Therefore, a certain amount of exposure seems necessary for herniation. Interestingly, increased cumulative exposure to daily lifting and standing/walking was not risk factors for receiving lateral re-operations (24). Therefore, after surgically closing the preformed defect in form of a patent processus vaginalis, factors other than increased intraabdominal pressure, or cumulative exposure to daily lifting and standing/walking, seem to cause lateral recurrences.

## Processus Vaginalis

Patent processus vaginalis is a risk factor for developing lateral hernias. Both persistent smooth muscle cells (25–29) and insufficient release of calcitonin gene-related peptide from the genitofemoral nerve may play a role in failed obliteration (30–32).

### The Relationship between a Patent Processus Vaginalis and Herniation

Processus vaginalis is formed by protrusion of peritoneum during the descensus of the testes, whereafter it should obliterate. Patency of the processus vaginalis and inguinal hernias are strongly correlated (33). The right testis descends later than the left (33), and both persistent processus vaginalis (34) and inguinal hernias are more frequent on the right side in term children (34, 35), whereas bilateral hernias are more common in preterm children (35). Also, patent processus vaginalis and inguinal hernias are more common in males (34–36). Asymptomatic patent processus vaginalis is reported in 20% of patients aged 5 months (34), 9% at 12 years (37), and 6–19% of adults (7, 36, 38). Thus, even though persistent processus vaginalis is a risk factor for developing a lateral hernia (7), it is evident that other factors determine if a hernia actually develops. However, it seems likely that a larger orifice of the proximal annulus would facilitate herniation.

### The Role of Smooth Muscle Cells and Molecules in Obliteration of Processus Vaginalis

Smooth muscle cells are proposed to have a function in testicular descent by “propelling the testis into the scrotum” (39) whereafter apoptosis of the cells may facilitate obliteration of processus vaginalis (26, 40). In fact, smooth muscle cells have been found to be more frequent in sacs from inguinal hernias than from hydrocele and undescended testes (25–27), and studies have found insufficient apoptosis (28) and absence of apoptotic nuclei (29) in smooth muscle cells from processus vaginalis. Furthermore, researchers have found smooth muscle cells in a local thickening around the internal ring of lateral hernias (41), which indicates that obliteration of processus vaginalis has been incomplete, and herniation may have occurred through a patent or reopened processus vaginalis (42). Failed apoptosis could be related to the sympathetic nervous system, which enhances smooth muscle growth (43) and maintenance of smooth muscle cells *in vitro* (44).

A review suggested that androgens indirectly regulate the descent of the testes by acting on the genitofemoral nerve, possibly with release of calcitonin gene-related peptide (45). Several *in vitro* studies have investigated this peptide (30–32). It induces obliteration of processus vaginalis by transforming epithelial cells to mesenchymal cell phenotype, but calcitonin gene-related peptide binding was only directly associated with the mesenchymal fibroblasts (30). Fibroblasts secrete hepatocyte growth factor (46, 47), and the effect of the calcitonin gene-related peptide could be mediated by this growth factor, which acts directly on the epithelial cells (30, 31). This theory has been tested on hernia sacs, but the peptide only increased the level of the growth factor in some samples (32). It is still unclear if deficient endogenous calcitonin gene-related peptide disrupts obliteration of processus vaginalis (32) or if obliteration is regulated through other mechanisms such as transforming growth factor β-1, which stimulates fibrosis (48). Detailed investigations in humans have been hindered due to issues with finding proper animal models with patent processus vaginalis (49).

## Connective Tissue Alteration

Individuals with inguinal hernias have altered connective tissue compared with controls regarding ratio of collagen fibers, fascia architecture, and level of enzymes involved in connective tissue homeostasis (50–56). When comparing hernia subtypes, patients with medial hernias seem to have a different abdominal fascia architecture and possibly both larger collagen degradation and affected properties of the transversalis fascia due to altered enzyme activity (57–61).

### Ratio of Connective Tissue in the Groin

Table 1 describes fibers and enzymes involved in connective tissue homeostasis. Collagen is the most abundant fiber in connective tissue (62) and the ratio and cross linkage between the thick type 1 and the thin type 3 fiber largely determine the tensile strength and the mechanical stability of connective tissue. A systematic review strongly suggested that patients with inguinal hernias have a lower collagen 1:3 ratio in the abdominal wall tissue compared with controls (50). Differences between hernia subtypes have been shown (63), but the overall evidence is insufficient (50). One study found significantly lower type 1 and total collagen combined with higher type 3 collagen for lateral hernias compared with controls (64). This would give thinner collagen fibers and may either contribute to hernia formation or be a consequence of the herniation (64). Furthermore, collagen has been shown to decrease in both the transversalis fascia and the rectus sheath with aging (65). In contrary to adults, no alterations in collagen subtypes 1:3 ratios have been observed in children (66, 67).

**Table 1:** Connective tissue components.

Fibers	
Collagen	
Type 1	Most common collagen type (62) and a thick fiber (68). Often as fibril in tendons (69) and with type 3 in dermis (70)
Type 3	Fibrillary collagen (thin fiber) (68), often together with type 1 (70). A major component of reticular fiber (assembles in delicate net) in relation to adipocytes and muscle cells and also in the basement membrane (71)
Type 4	Filamentous network (does not assembles in fibers), in the basement membrane (72)
Type 5	Minor component of the extracellular matrix. Involved in the fibril-forming of collagen (73) and can be found between type 1 and 3 collagen (74)
Elastic fiber	Elastin is a protein and the major component of elastic fibers together with microfibrils (71). Elastin comprises of cross-linked tropoelastin (75)

**Enzymes**	

Lysyl oxidase	Catalyze the formation of elastin and cross-links collagen (62)
Matrix metalloproteinases	Zink-dependent enzymes (76)
Type 1, 13	Degrade collagen 1, 2, and 3 (among others) (76, 77)
Type 2, 9	Degrade collagen 1, 4, 5, and elastin (among others) (76, 77)

### Architecture and Mechanical Properties of Abdominal Fasciae

Comparing patients with inguinal hernias with controls have revealed both altered architecture of fascia fibers (51) and no difference (52). Comparing hernia subtypes, significantly less collagen and more abundant and disorganized elastic fibers have been shown in patients with medial hernias (57). Young men with medial hernias have also been shown to have thinner rectus sheaths compared with men with lateral hernias and controls (58). A study on mechanical properties of the transversalis fascia found no difference in inguinal hernias compared with controls, and the fascia expansion in medial hernias may solely be a response to the mechanical pressure from the hernia (78). Since cumulative intraabdominal pressure does not increase the risk of primary medial repairs (11, 12), it is unclear which factors could promote herniation in the absence of a weakened transversalis fascia.

### Enzymes Potentially Involved in Inguinal Hernia Development

Two types of enzymes have been investigated for their role in inguinal hernia development: matrix metalloproteinases (MMPs), which digest proteins of the extracellular matrix to maintain tissue homeostasis (76, 77), and lysyl oxidase, which cross-links collagen and elastin (59). Their influences on hernia development are not fully understood, but an increased activity of MMPs could explain the altered collagen ratios seen in inguinal hernias, and a decreased activity of lysyl oxidase would affect the elastic and mechanical strength of connective tissue. Significantly higher levels of MMP-1, -2, and -9 in the transversalis fascia have been shown in patients with inguinal hernias versus controls (53). Higher levels of MMP-2 have been shown in patients with medial hernias versus lateral hernias (60, 61), which may be due to activation by the cytokine transforming growth factor β-1 (79). For lysyl oxidase, significantly lower levels of the enzyme combined with higher elastase activity have been shown in medial hernias compared with lateral hernias and controls, and impaired elastic property of the transversalis fascia may especially contribute to medial hernia formation (59). Since the enzyme is copper dependent, lower levels of copper would theoretically lower its activity. Lower levels of copper have been shown in the transversalis fascia of patients with inguinal hernias (54) and when comparing hernia subtypes, both different (80) and equal (54) copper levels have been found.

### Systemic Alteration of Connective Tissue in Patients with Inguinal Hernias

Inguinal hernias are common in patients with connective tissue disorders, and several studies have investigated if hernia is a local phenomenon of a systemic connective tissue imbalance. Significantly lower collagen subtype 1:3 ratio in skin has been shown for individuals with inguinal hernias (52, 55) without any differences between hernia subtypes or MMP-1/13 levels (55). Higher active MMP-2 levels have been found in abdominal skin of patients with medial hernias versus controls, without a difference between hernia subtypes, implying different levels in MMP-modulators (81). Finally, researchers have found a reduced turnover of the interstitial collagen types 3 and 5 and an increased turnover of the basement membrane collagen type 4 in the blood of patients with inguinal hernias, which suggests a systemic imbalance between interstitial- and basement membrane matrices (56). With these systemic findings, altered connective tissue in patients with inguinal hernias must be more than a local response to mechanical pressure from the hernia and thereby play a potential role in the hernia etiology.

## Genetics

Inguinal hernias are hereditary with a complex multifactorial inheritance pattern (82, 83). A nationwide study found that groin hernias are clustered in families, which was most prominent for daughters to mothers that had undergone groin hernia surgery (84). Family specific mutations have been identified in a family with lateral and medial hernias through several generations (85). Generalizable mutations for patients with inguinal hernia have been investigated (86–89), and researchers have recently found four inguinal hernia susceptible loci that seem to be involved in connective tissue homeostasis (90).

## Discussion

Lateral and medial hernias seem to have both common and different etiologies, and the risk factors are summarized in Table 2. A patent processus vaginalis and increased cumulative mechanical exposure are risk factors for lateral hernias. Medial hernias seem to have a more profoundly altered connective tissue architecture and homeostasis compared with lateral hernias. However, altered collagen ratios are seen for both hernia types in adults, and combined with the peak prevalence of hernias observed late in life, connective tissue alterations may very well play a role in development of both subtypes. Furthermore, inguinal hernias have a hereditary component with a complex inheritance pattern, and inguinal hernia susceptible genes have been identified that also are involved in connective tissue homeostasis.

**Table 2:** Possible etiological factors.

	Lateral hernia	Medial hernia	Results	Reference
**External risk factors**				
High intraabdominal pressure	+	−	Increase cumulative occupational mechanical exposure increase the risk of lateral hernia repairs, but not lateral re-operations	(11, 12, 24)
Smoking	?	?	May theoretically increase herniation, but this has not been confirmed	(10, 12, 20, 21)
**Patient risk factors**				
Age	+	+	Increasing age increase the risk of both lateral- and medial repairs	(9, 10, 12)
Connective tissue alteration	+	+	Both medial and lateral hernias have altered connective tissue compared with controls. Medial hernias seem to have a more profound alteration	(50–61)
Connective tissue disorders	+	+	This is a shown risk factor for inguinal hernias, but studies have not subdivided the results on hernia type	(13, 14)
Constipation	−	−	Does not appear to be a risk factor	(17)
Genetics	+	+	Gene mutations are reported for both hernia types, and generalizable mutations for inguinal hernias are possibly identified	(82–90)
Low body mass index (BMI)	+	+	A higher BMI is a protective factor	(10, 12, 15)
Male gender	+	+	Studies report male gender as a risk factor for inguinal hernias, without subdividing the results on hernia type	(9, 10)
Patent processus vaginalis	+	−	A risk factor, but not all patients with a patent processus vaginalis develop a lateral hernia. The exact mechanism why processus vaginalis fails to obliterate is not established	(7, 25–32, 34, 36–38)
Prostatic hypertrophy	?	?	A weak association has been found	(17–19)

*+, a risk factor; −, not a risk factor; ?, unknown if it is a risk factor*.

### Prevention of a Primary Inguinal Hernia

We lack diagnostic tools that can predict who will develop an inguinal hernia. Even though gene tests may be used in the future to predict who is at risk, surgical repair of an asymptomatic inguinal hernia may cause more harm than benefit since 10–12% of patients develop chronic groin pain after operation (91, 92). Laparoscopic repairs seem most promising regarding chronic pain (93), but the difference between laparoscopic- and the Lichtenstein repair seems to equalize after 3–4 years (94, 95). Gene tests are thereby only indicated if the chronic pain rate can be lowered after surgery. Even though some professions increase the risk of lateral hernia repairs (11, 12), there is no need to discuss inguinal hernias in employment counseling since many will never develop a hernia. However, if a drug was developed that could close processus vaginalis, it should specifically be considered for individuals with a physically demanding job or for patients identified at risk by gene analysis. Therefore, further research on a potential role of calcitonin gene-related peptide deficiency and/or other mediators is warranted.

### Incorporation of Hernia Etiology in Precision-Based Medicine

The definition of precision-based medicine is to “seek to improve stratification and timing of preventive and therapeutic measures by utilizing biological information and biomarkers on the level of molecular disease pathways, genetics, proteomics as well as metabolomics” (96). Today, we lack diagnostic tests that allow incorporation of the etiology into the concept of precision-based medicine. If biomarker tests could reveal contributing factors to hernia development such as disturbances in the collagen profile, or properly map the gene profile for medial, lateral, bilateral, and recurrent inguinal hernias, then the possibilities for tailored surgery would expand.

Surgical techniques are needed that lower the chronic pain rate after inguinal hernia surgery, and based on the differences in the etiology, tailored surgery for the two hernia subtypes should be considered. One way to lower chronic pain might be to leave less material in the groin, which can be accomplished by using absorbable meshes or by performing sutured repairs.

It is important that absorbable meshes are replaced by new connective tissue to prevent recurrences and these meshes should probably only be considered for lateral hernias (97). However, two systematic reviews have shown no difference in the chronic pain rate between absorbable and permanent meshes, but there were insufficient studies to make a solid conclusion (97, 98).

Regarding performing sutured repairs, guidelines have discussed if it would be beneficial for young patients with lateral hernias (94, 99) since younger individuals have a higher risk of developing chronic postoperative pain (100, 101). A recent study showed that 18- to 29-year-old males had a significantly lower cumulated re-operation rate after sutured repair compared with 30- to 99-year-old males (102). It is unclear if sutured repairs actually lower the chronic pain rate in this group, but if that would be the case, sutured repairs could be a valid alternative for young men with lateral hernias.

Permanent meshes seem essential for patients with medial hernias due to the more profound connective tissue alteration. Animal studies have assessed the effect of adding stem cells to meshes with somewhat promising results (103, 104). Hopefully, stem cell-coated meshes could be a method to lower the recurrence rate after medial hernia repairs, and perhaps be considered for individuals with a family history of inguinal hernias due to the increased risk of earlier recurrences (82).

### Clinical Perspective

Based on this review, we can answer some of the questions frequently posed by patients. Could the patient have prevented the inguinal hernia? The simple answer is no, unless they have a lateral hernia and a physically demanding job (11, 12), but the increased risk disappears after repair (24). Patients might also ask if training of the abdominal wall can prevent a primary hernia or lower the risk of a recurrence. Data are lacking to support this, but very skinny patients can slightly increase their BMI to reduce the risk of a primary hernia to occur (10, 12, 15), and patients with a high BMI should lose some weight to prevent a recurrence (4). About 11% of patients with a primary inguinal hernia will have a contralateral repair within 10 years (105). A question surgeons may ask is if we should operate bilaterally instantly. Due to the 10–12% risk of developing chronic pain, the answer is no. However, future biomarker tests may change this.

### Conclusion

Medial and lateral hernias both have common and different etiologies. Risk factors to develop both lateral and medial hernias are older age, a low BMI, and gene mutations. Even though connective tissue alteration is confirmed in both hernia subtypes, medial hernias appear to have a more profound alteration. Patent processus vaginalis and increased cumulative occupational mechanical exposure are risk factors to develop lateral hernias.

## Author Contributions

SÖ contributed substantially to the conception and design of the work, the acquisition and interpretation of data, and drafted the work. KA and JR contributed substantially to the conception and design of the work, the interpretation of data, and revised the work critically for important intellectual content. All authors have approved the final version to be published and have agreed to be accountable for all aspects of the work in ensuring that questions related to the accuracy or integrity of any part of the work are appropriately investigated and resolved.

## Conflict of Interest Statement

SÖ reports no potential conflicts of interest. KA reports personal fees from Bard outside the submitted work. JR reports personal fees from Bard and Merck, outside the submitted work.
